# All laser direct writing process for temperature sensor based on graphene and silver

**DOI:** 10.1007/s12200-024-00108-4

**Published:** 2024-02-05

**Authors:** Qi Li, Ruijie Bai, Lianbo Guo, Yang Gao

**Affiliations:** 1 Shanghai Key Laboratory of Intelligent Sensing and Detection Technology, School of Mechanical and Power Engineering, East China University of Science and Technology, Shanghai, 200237 China; 2 North Automatic Control Technology Institute, Taiyuan, 030006 China; 3 Wuhan National Laboratory for Optoelectronics, Huazhong University of Science & Technology, Wuhan, 430074 China

**Keywords:** Laser direct writing, Temperature sensor, Finite element analysis, Laser induced graphene, Laser induced silver

## Abstract

A highly sensitive temperature sensing array is prepared by all laser direct writing (LDW) method, using laser induced silver (LIS) as electrodes and laser induced graphene (LIG) as temperature sensing layer. A finite element analysis (FEA) photothermal model incorporating a phase transition mechanism is developed to investigate the relationship between laser parameters and LIG properties, providing guidance for laser processing parameters selection with laser power of 1–5 W and laser scanning speed (greater than 50 mm/s). The deviation of simulation and experimental data for widths and thickness of LIG are less than 5% and 9%, respectively. The electrical properties and temperature responsiveness of LIG are also studied. By changing the laser process parameters, the thickness of the LIG ablation grooves can be in the range of 30–120 μm and the resistivity of LIG can be regulated within the range of 0.031–67.2 Ω·m. The percentage temperature coefficient of resistance (TCR) is calculated as − 0.58%/°C. Furthermore, the FEA photothermal model is studied through experiments and simulations data regarding LIS, and the average deviation between experiment and simulation is less than 5%. The LIS sensing samples have a thickness of about 14 μm, an electrical resistivity of 0.0001–100 Ω·m is insensitive to temperature and pressure stimuli. Moreover, for a LIS-LIG based temperature sensing array, a correction factor is introduced to compensate for the LIG temperature sensing being disturbed by pressure stimuli, the temperature measurement difference is decreased from 11.2 to 2.6 °C, indicating good accuracy for temperature measurement.

**Graphical Abstract:**

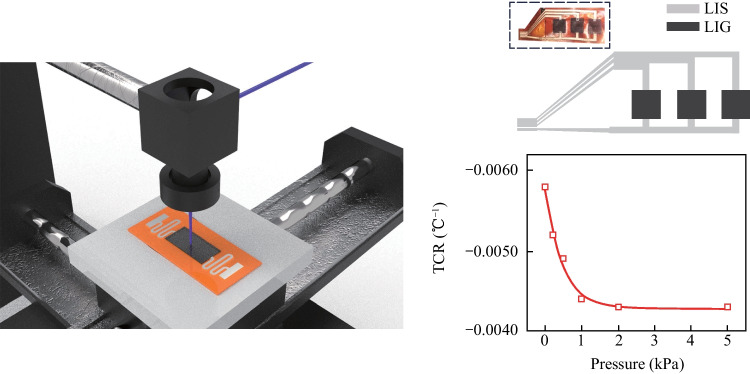

**Supplementary Information:**

The online version contains supplementary material available at 10.1007/s12200-024-00108-4.

## Introduction

In recent years, there has been rapid development of 5G technology, the Internet of Things (IoT), artificial intelligence and human machine interfaces. To match some of these developments, scalable fabrication methods for flexible sensors are in high demand. Currently, the common preparation processes, including vapor deposition [1], photolithography [2–4], inkjet printing [5] and screen printing [6, 7], can fabricate sensors in large scale. However, these methods have some significant defects. For instance, chemical and physical vapor deposition [1, 8] methods can produce high-performance sensing device, but the strict fabrication conditions are required. Photolithography [2] offers high fabrication accuracy for electronics, while the poor compatibility for materials leads to a complex manufacturing process. Inkjet printing technology [5] involves a simple process and allows direct patterning, however the ink preparation process is time-consuming. Therefore, an effective, compatible and compact fabrication method for flexible sensors is still required.

Laser direct writing (LDW) method, utilizing the photo-thermal conversion, can synthesize materials and then engrave them with the desired morphologies and structures [9–12]. This versatile process has been widely employed to reduce procedures [13], including metal salt solution [11, 14, 15] (Ag, Cu, Pd, etc.) and carbon (graphene oxide, Mxene) [9, 10, 16, 17]. Currently, numerous applications of LDW technology have been reported for flexible sensor manufacturing of various types, such as for temperature [18, 19], mechanical [20–22], acoustic [23, 24], optical [25–27], gas [28, 29] and biosensor [30, 31] detection. To name a few, Shin and coauthors [18] demonstrate the Ni/NiO based temperature sensor through LDW method. The NiO layer is utilized as the temperature sensing material due to its good response to temperature change. The Ni layer is sintered from NiO nanoparticles and used as electrodes. The sensor exhibits high TCR value of 0.4%/℃ [18]. Liu and coauthors present a patternable pressure sensor based on laser induced graphene (LIG), using a CO_2_ laser. The highest gauge factor of the pressure sensor achieves up to 112 [32]. Samouco et al. [33] create a UV sensor with high sensitivity by simple LDW and drop-casting methods. Pinheiro et al. [34] investigate the impact of laser irradiation parameters on electrical and electrochemical properties of paper-based graphene, for modulation the performance of LIG-based electrochemical three-electrode cells. Sun and coauthors prepare a humidity sensing device based on reduced graphene oxide (rGO) by a two-beam-laser interference mediated method. When the relative humidity increases from 11% to 95% at room temperature, the resistance of the device can be reduced to 3% of its initial value, with the response time of 3 s and recovery time of 55 s [35]. However, there are still several critical challenges to be addressed for LDW methods. For example, most of the aforementioned studies are specifically focused on the single functional materials, while researches on sensors with different types of materials by LDW methods are lack. Moreover, the photo-thermal effect induced by laser may lead to the change of material properties such as morphology and electrical performance; this should be investigated more thoroughly.

Herein, a highly sensitive temperature sensing array is prepared by all LDW method, using laser induced silver (LIS) as electrodes and LIG as the temperature sensing layer. To address the relationship between LDW photo-thermal conversion and morphology structure of LIG and LIS, a finite element analysis (FEA) photothermal model incorporating a phase transition mechanism is developed. The deviation of simulation and experiment data for widths and thickness of LIG samples is less than 5% and 9%, which demonstrates the accuracy of the FEA model. According to the FEA calculation result, the laser power and laser scanning speed are selected as 1–5 W and greater than 50 mm/s, respectively. By changing the laser process parameters, the thickness of the LIG can be controlled in the range of 30–120 μm, and the resistivity of LIG can be regulated within the range of 0.031–67.2 Ω·m and the TCR is calculated as − 0.58%/°C. Furthermore, the FEA photothermal used in the experiments and simulations, with average experiment-simulation difference less than 5%. The suitable laser power and scanning speed are fixed at the laser scanning rate of 50 mm/s and the laser power of 1.5–3 W. The LIS sensing samples have a thickness of about 14 μm, resistivity of 0.0001–100 Ω·m, which are insensitive to temperature and pressure stimuli. Since the temperature sensing is disturbed by pressure stimuli, by introducing the correction factor, the temperature calculation deviation is decreased from 11.2 to 2.6 °C, indicating the good accuracy for temperature measurement.

## Experimental section

### Materials

Polyimide (PI) and ethanol ((C_2_H_5_OH)_*n*_, AR) are purchased from Shanghai Aladdin Biochemical Technology Co., Ltd. Silver nitrate (AgNO_3_), sodium citrate tribasic dihydrate (99.5%) and polyvinylpyrrolidone (PVP, AR) are purchased from Sinopharm Chemical Reagent Co., Ltd. The above reagents are used as received without further purification.

### Synthesis of the LIG

The 355 nm pulsed wave laser (MMEPU-355-5, Tianjin Meiman Laser Technology Co., LTD, beam diameter = 30–70 μm) is utilized to synthesize LIG by carbonizing PI substrate (the thickness of 250 μm) in ambient environment. The laser power and scanning speed are changed from 1 to 5 W and 10 to 200 mm/s, respectively, and the laser irradiation area is rectangular with a size of 10 mm × 5 mm, with five samples included in each set of parameters to verify the reproducibility.

### Synthesis of the LIS

The 1.0 g sodium citrate tribasic dihydrate and 100 mg PVP are mixed in 25 mL deionized water. The 1.72 g AgNO_3_ is dissolved in 25 mL deionized water. Then, the above two solutions are mixed and stirred completely at room temperature for one hour to get the LIS precursor. Specifically, PVP is utilized as a dispersant, which can prevent the agglomeration of tri-silver citrate and improve the wettability of precursor. The LIS precursor is uniformly coated on the surface of PI film, and dried at 60 °C for four hours to form a layer of 1 mm thickness. A 355 nm continuous wave laser is used to synthesize LIS in ambient environment. The laser power and scanning speed are changed from 1 to 3.5 W and 20 to 50 mm/s, respectively, and the size of irradiation area is 10 mm × 5 mm, and five samples are prepared for each group of parameters. After processing, the redundant silver paste can be removed by laser ablation.

### Characterization of LIG and LIS morphology

The morphology investigation for LIG and LIS is performed by the field emission scanning electron microscope (FESEM, Hitachi, S4800). The chemical compositions of materials are investigated by energy dispersive X-ray spectroscopy (EDX, HAL100, ZEISS, Co., LTD).

### Characterization LIG and LIS electromechanical performance

A digital source meter (34972A, Keysight) is used to measure the electrical response caused by applied pressures. The sensitivity (*S*) for the pressure sensing is estimated by

1 $$S = \frac{{{{\Delta R_{{\text{p}}} } \mathord{\left/ {\vphantom {{\Delta R_{{\text{p}}} } {R_{{{\text{po}}}} }}} \right. \kern-0pt} {R_{{{\text{po}}}} }}}}{\Delta P},$$

where ∆*P* is the pressure experienced by the device; *R*_po_ and ∆*R*_p_ are the initial resistance and resistance variation for the device, respectively.

The electrical response induced by the temperature change is examined by the electrochemical station (CHI660E, Shanghai Chenhua Instrument). The TCR of the device is evaluated via

2 $${\text{TCR}} = \frac{{R_{T} - R_{T_0} }}{{R_{T_0} \cdot (T - T_{0} )}} \times 100\% ,$$

where $R_{T_0}$ and *R*_*T*_ are the resistances of the device at room temperature (*T*_0_ ~ 24 °C) and tested temperature of *T*, respectively. The above experiments are conducted under conventional atmospheric conditions (25 °C, 65 RH%).

## Results and discussion

### FEA model for LIG carbonization process

**Fig. 1:**
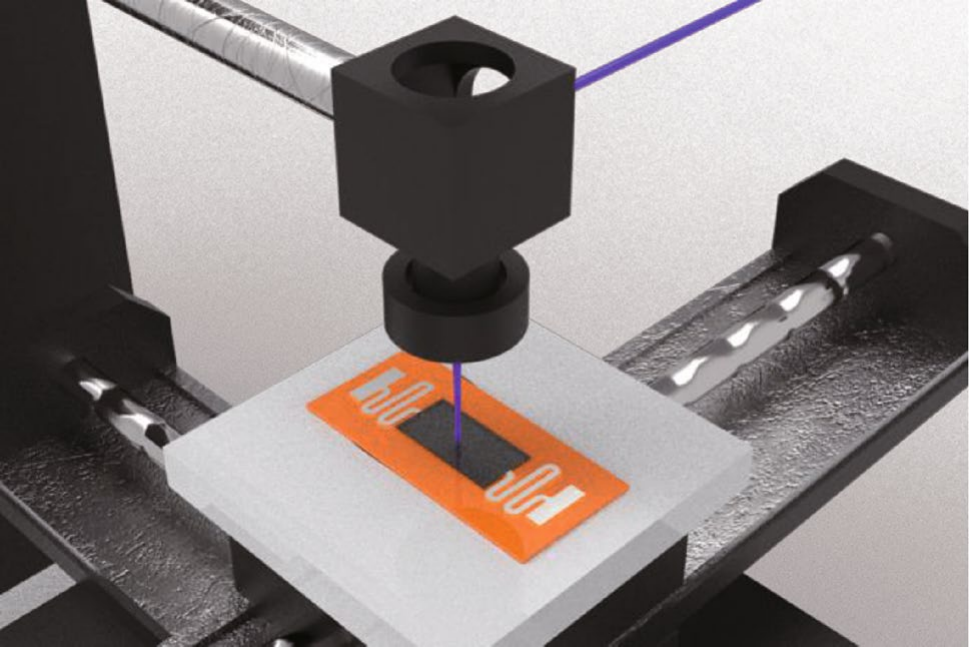
Schematic illustration of the LDW process for fabrication of an LIG-LIS based temperature sensor

The LIG carbonization process is described as follows: when the laser beam irradiates the PI surface, the laser reflection occurs at the interface of PI and air (as shown in Fig. 1). At the same time, the un-reflected light of the laser enters into the PI film and is gradually absorbed. The absorption coefficient of PI film can be written as *α* and the reflectivity of PI as *β*. The laser beam directly raises the temperature of irradiated area, and the temperature outside the irradiated area is raised by heat conduction.

Based on the energy distribution of the laser beam, satisfying the Gaussian characteristic, and the reflection loss occurring at the PI/air interface, conforming to the Beer-Lambert law, and using a cylindrical coordinate system, then the heat source density per unit volume at a position (*r*,* z*) is denoted as

3 $${q}(r,z) = \alpha (1 - \beta )\frac{2P}{{{\uppi }w_0^{2} }}\exp \left[\frac{{ - 2r^{2} }}{{w(z)^{2} }}\right]\exp ( - \alpha z),$$

4 $$r = \sqrt {(x - vt)^{2} + y^{2} } ,$$

5 $$w(z) = w_{0} \sqrt {1 + \left( {\frac{z}{{z_{{\text{R}}} }}} \right)^{2} } ,$$

6 $$z_{{\text{R}}} = \frac{{{\uppi }w_{0}^{2} }}{{\lambda_{0} }},$$

where *r* is the radial distance away from the laser beam centerline and changes with the movement of laser beam center; *z* is the vertical distance from the PI surface; *w*(*z*) is the waist variation of laser beam in vertical direction; *z*_R_ is the parameter correlated with the waist and wavelength of the laser beam; *P* is the laser power and *w*_0_ is the waist of laser beam.

Due to the increase of temperature during the irradiation process, the properties of PI film are changed by the phase transition. The mechanism equation is expressed as

7 $$C_{{\text{p}}} = \theta_{1} C_{{{\text{p}},{1}}} + \theta_{2} C_{{{\text{p}},{2}}} + L_{1 \to 2} \frac{{\partial \alpha_{{\text{m}}} }}{\partial T},$$

8 $$\alpha_{{\text{m}}} = \frac{1}{2}\frac{{\theta_{2} - \theta_{1} }}{{\theta_{2} + \theta_{1} }},$$

9 $$\kappa = \theta_{2} \kappa_{2} + \theta_{1} \kappa_{1},$$

10 $$\theta_{1} + \theta_{2} = 1,$$

where *θ*_1_ represents the proportion of initial phase; *θ*_2_ denotes the proportion of transition phase and *L*_1→2_ is set as the temperature of phase transition. *C*_p,1_, *C*_p,2_, *κ*_1_, and *κ*_2_ represent the specific heat capacities and the absorption coefficients for phase 1 and phase 2, respectively.

Combining the previous equations with the classical heat transfer equation, a transient heat equation for the temperature distribution *T* (*r*, *z*, *t*) can be written as

11 $$\rho C_{{\text{p}}} \frac{\partial T}{{\partial t}} - \nabla k\nabla T = q(r,z).$$

With the corresponding initial condition:

12 $$T(r,z;t = 0) = T_{{{\text{ext}}}} = 293\,{\text{K}}.$$

And boundary condition:

13 $$\overrightarrow {n}\cdot \overrightarrow {q} = h_{1} (T_{{{\text{ext}}}} - T),\quad {\text{for top of surface,}}$$

14 $$\overrightarrow {n}\cdot \overrightarrow {q} = h_{2} (T_{{{\text{ext}}}} - T),\quad {\text{for bottom of surface}},$$

15 $$\overrightarrow {n} \cdot\overrightarrow {q} = 0,\quad {\text{for edges}},$$

where the upper and lower surfaces of the PI film are natural convection boundaries, and the heat transfer coefficients *h*_1_ and *h*_2_ are derived from the empirical functions. Since the lateral size of the PI film in the model is much larger than the laser spot area, the boundary conditions are set as thermal isolation boundary conditions. It should be specifically noted that the heat released and absorbed by the PI film itself is neglected in this model. The heat released and absorbed by PI carbonization can reach 755 J/g, while for laser body heat source, the heat input energy within the laser irradiation area is normally in the range of 10^6^–10^8^ J/g. Therefore, the heat released and absorbed by PI itself is much smaller than the energy input from the laser direct writing, and can be ignored in this model.

**Fig. 2:**
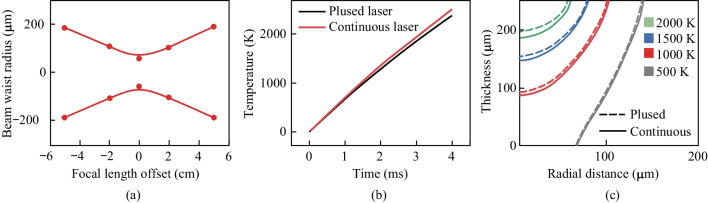
**a** Focal length offset versus beam waist radius curve. **b** Irradiation area temperature and **c** isothermals for continuous and pulsed laser in 0–2000 K

Figure 2a shows the experimental focal length offset versus beam waist radius. Accordingly, it can be assumed that the beam waist radius of 355 nm pulsed wave laser is 48 µm. Figure 2b, c show the comparison of irradiation area temperature and isothermals for continuous and pulsed laser, respectively. As shown in Fig. 2b, the spot center temperatures (< 1000 K) of the pulsed and continuous laser beam have very small difference. When the temperature is near 2500 K, the difference is less than 3%. As for isothermals curves of continuous and pulsed laser (in Fig. 2c), there is a slightly difference for 2000 K isothermals curves, exhibiting the difference of 5 µm in transverse dimension and 15 µm in longitudinal dimension. Moreover, the isotherm curves at 1000 K demonstrate differences of < 2 µm in transverse dimension and < 5 µm in radial dimension. Thus, it can be concluded that in high-temperature region (> 2000 K), the temperature resulting from the continuous-light simulation is higher than that from the pulsed-light simulation, but the difference is not greater than 5%. In the low temperature region (< 1000 K), the use of continuous or pulsed light has negligible effect on simulation results. Therefore, the pulsed laser in FEA model is substituted by the continuous laser, which can significantly decrease the calculation complexity.

### Characterization of LDW parameters for LIG morphology

**Fig. 3:**
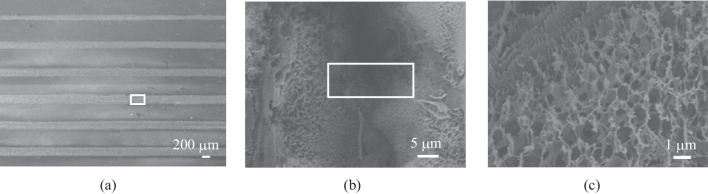
**a** SEM image of LIG. **b**, **c** Magnified SEM images for LIG microstructures

**Fig. 4:**
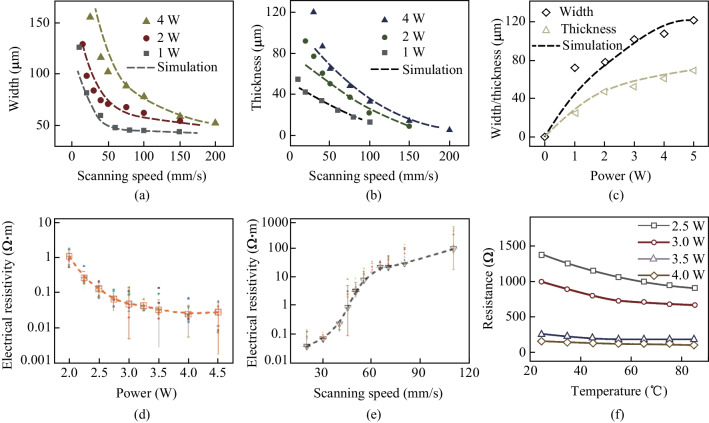
Comparison of simulation and experimental data of: **a** LIG ablation grooves widths for different laser scanning speeds; **b** LIG ablation grooves thickness for different laser scanning speeds; **c** LIG ablation grooves widths and thickness with different laser powers, respectively. The electrical resistivity curves of: **d** LIG samples generated at different laser powers; **e** LIG samples generated at different laser scanning speeds; **f** temperature–resistance curves for the LIG samples generated at different laser powers with the scanning speed of 50 mm/s

The FEA model is utilized to investigate the relationship between photo-thermal conversion during the LDW process and morphology structure of LIG. In order to validate the validity of FEA model, the LIG samples are generated at different laser powers and scanning speeds. Figure 3a exhibits the SEM images of laser processing grooves for LIG, and Fig. 3b, c show the porous structures created by the release of oxygen-containing gases in the LDW process. The generated LIG mainly includes C, N and H elements, and the ratio of C element satisfies the Gaussian distribution, which has the highest level at the irradiation center (Fig. S1). The comparison between FEA calculation results and experimental data is plotted in Fig. 4a–c. As shown in Fig. 4a, the ablation groove widths for calculation results achieve good agreement. When the laser power is fixed in the range of 1–4 W, with the laser scanning speed higher than 50 mm/s, the experiment-simulation difference for calculation of width is no more than 3%. However, with the decrease of laser scanning speed, the experiment-simulation difference for calculation of widths increases gradually. For instance, when the laser scanning speed is about 10 mm/s, the experiment-simulation difference in width values is more than 8%. Figure 4b shows the ablation groove thickness for different laser scanning speeds with the laser power of 1–4 W. With the laser scanning speed greater than 50 mm/s, the experiment-simulation agreement regarding ablation groove thickness is good and the deviation is no more than 4%. Moreover, experiment-simulation difference for the LIG ablation grooves prepared by the constant laser scanning speed of 50 mm/s with different laser powers are investigated (Fig. 4c). The average experiment-simulation difference for thickness is no more than 5%, while that for widths is approximately 9%. Overall, for higher laser scanning speed and constant laser power of 1–4 W, the FEA model exhibits the good experiment-simulation agreement and the differences are acceptable, providing guidance for laser processing parameters adjustment.

### Electromechanical characterization of LDW parameters for LIG

The electrical resistivity of LIG is measured by a four-probe resistivity meter. Each sample is measured more than five times and an average is used to avoid excessive influence of any abnormal values. Figure 4d shows the electrical resistivity curves for LIG samples generated at different laser powers. Notably, the increase of laser power, from 2 to 3 W, leads to a remarkable decrease in electrical resistivity (from the initial 1.07 to 0.054 Ω·m), which is a drop of nearly 95%. However, with the further increase of laser power, from 3 to 4.5 W, the electrical resistivity experiences a smaller decline from 0.054 to 0.031 Ω·m. When the laser power is raised from 4 to 4.5 W, electrical resistivities show negligible change. Figure 4e shows the electrical resistivity curves for LIG samples generated at different laser scanning speeds. When the laser scanning rate decreases from 80 to 40 mm/s, the electrical resistivity decreases from 67.2 to 0.279 Ω·m. However, when the scanning rate is further reduced from 40 to 20 mm/s, the electrical resistivity is only decreased from 0.279 to 0.087 Ω·m. It is suggested that much higher laser power or much lower laser scanning speed leads to the high input energy density, which causes over-carbonization and LIG structural damage, restricting the further decrease of electrical resistivity.

Figure 4f shows the temperature-resistance curves for the LIG samples generated at different laser powers, with the scanning speed of 50 mm/s. The LIG samples demonstrate the negative TCR, due to increase of temperature causing the decrease of resistance. The TCR values are calculated as − 0.0058, − 0.0055, − 0.0052, and − 0.0049 °C^−1^ for the laser powers of 2.5, 3.0, 3.5, and 4.0 W, respectively. The samples prepared at lower laser power exhibit higher TCR compared to those prepared at higher laser power. The comparison of temperature sensing performance for LIG with representative examples is listed in Table S1. The device is in ambient atmosphere (25 °C ± 2 °C, 65% RH) for two weeks, and then the temperature responsiveness is measured for 80 °C both before and after aging test. The results in Fig. S2 reveal minimal changes in temperature response, highlighting the device’s stability.

### Determination of LDW parameters for LIS morphology

**Fig. 5:**
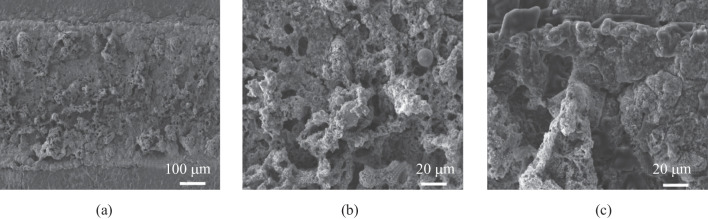
**a** SEM image of LIS based grooves. **b**, **c** Magnified SEM images of LIS based grooves

Due to the electrical properties of LIS, electrodes performance are influenced by morphology of particles. The FEA simulation is used to investigate the LDW parameters for LIS. Figure 5a shows LIS grooves with the clear boundary between the reduced and unreduced areas. Figure 5b and c show the enlarged images of the center areas, indicating the silver particles aggregating in small spheres. The porous structures are distributed over the LIS surfaces, which is attributed to the gases such as nitrogen and carbon dioxide, during the thermal reduction process. A higher porosity in the center region indicates a good reduction of the silver precursor; therefore, the lower porosity in the edge region represents the uncomplete reduction for LIS. The EDS that scans along the vertical width direction is employed to analyze the element composition of the LIS samples. Figure S3 displays the EDS spectrum for an LIS sample. Points 1 and 7 are located at the unreduced region with approximately 30% silver, which is much lower than the theoretical mass fraction. As for points approaching the center of the laser scanning path, the Ag content increases gradually and mass fraction becomes close to 90%, indicating that good thermal reduction occurred. At the same time, the O, N and Na element contents decrease sharply.

To further investigate the relationship between laser processing parameters and LIS morphology, FEA models are established using silver precursors on the PI substrate. The specific parameters of silver precursors including light absorption coefficient, thermal conductivity and specific heat capacity are determined as follows:

1. Absorption coefficient
  The UV spectroscopy method is used and the optical behaviors at different wavelengths are illustrated in Fig. S4. The absorption coefficient of silver precursor is calculated as 1.35 × 10^4^ m^−1^.
2. Specific heat capacity
  Figure S5 illustrates the specific heat capacity versus temperature curve. The specific heat capacity increases gradually from 0.23 to 0.30 J/(g·K) for temperature below 100 °C. After the temperature reaches 300 °C, the specific heat capacity attains approximately 0.31 J/(g·K). For much higher temperature, the specific heat capacity demonstrates a slight decline to 0.28 J/(g·K) and then remains stable.
3. Thermal conductivity
  Thermal conductivity is usually measured by the film thermal conductivity analyzer. The thermal diffusion coefficient for Ag precursor is taken as 60.13 W/(K·m).

**Fig. 6:**
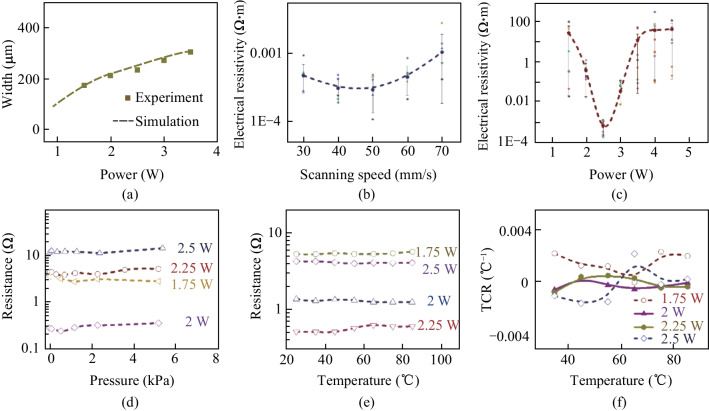
**a** Comparison between experimental and simulation results for region widths of LIS samples generated at laser power of 1–3.5 W and laser scanning speed of 50 mm/s. The electrical resistivity for LIS samples versus, **b** different laser scanning speeds, and **c** different laser powers. The resistance response of the LIS samples under **d** different pressures, and **e** different temperatures. **f** TCR curves of LIS samples under different temperatures

The FEA simulation for LIS is similar to that used in the aforementioned LIG process. Figure 6a exhibits the comparison between experimental region widths and simulation results for LIS samples generated at laser power of 1–3.5 W and laser scanning speed of 50 mm/s. Overall, the calculation results are a good fit with the experimental results, with difference less than 5%.

### Determination of LDW parameters for LIS

Figure 6b shows the electrical resistivity of LIS samples versus different laser scanning speeds. The electrical resistivity values remain in the range of 0.0001–0.001 Ω·m. In Fig. 6c, the electrical resistivity versus laser power for LIS samples is plotted. As for the laser power of 2.5 W, the electrical resistivity is calculated within the range of 0.0001–0.002 Ω·m. However, for much higher or extra lower laser power, such as 4.5 and 1 W, the electrical resistivity values are seen to be in the range of 10–100 Ω·m. Therefore, for the aforementioned FEA simulation, the LIS fabrication conditions are fixed at the laser scanning rate of 50 mm/s and laser power of 1.5–3 W. Figure 6d shows the resistance response of the LIS samples under different pressures. The resistance change of the LIS samples is no more than 10% in the pressure range of 0–8 kPa, which indicate the low sensitivity to pressure stimuli. Figure 6e shows resistance response of the LIS samples at different temperatures. In the temperature range of 20–80 °C, the LIS samples exhibit slight fluctuations in resistance. To visualize the response to temperature, the TCR of LIS samples prepared at different powers are listed in Fig. 6f. The TCR of samples prepared at 2 and 2.25 W are consistently below 0.001 °C^−1^. Conversely, the TCR of samples prepared at lower power (particularly at 1.75 W) is relatively large, with the maximum value of 0.0025 °C^−1^ at low temperatures.

### Determination of LIS-LIG based temperature sensing array by the LDW process

**Fig. 7:**
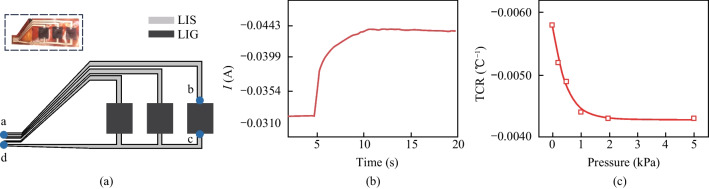
**a** Schematic of 1 × 3 temperature sensor array fabricated by LDW method. **b** *I*–*t* curve of a cylinder with the weight of 5 g at 80 °C put on the sensor array. **c** TCR for LIG under the pressure load of 0–5 kPa

Figure 7a shows a schematic of a 1 × 3 temperature sensor array fabricated by the LDW method, with LIS electrodes and LIG sensing layer. In detail, the laser processing parameters for LIS use laser power of 2.5 W and laser scanning speed of 50 mm/s. Meanwhile, as for the generation of LIG, the laser power is fixed at 2.5 W and the laser spanning rate is 50 mm/s. The resistances between points a–b, b–c, and c–d are measured as 0.33, 21, and 0.14 Ω, respectively. Notably, the resistance value of LIS is only 2.35% of LIG, indicating the good conductivity for electrical connection.

To validate the performance of the temperature sensing array, a cylinder with a mass of 5 g is positioned on the device, at 80 °C. The *I − t* curve is depicted in Fig. 7b, which indicates a rapid current increase firstly and then stability after approximately 5 s. This phenomenon is attributed to decrease of resistance of the LIG with the increase of temperature. The initial current, denoted as *I*_0_, is measured as 0.0322 A. While for the stabilized baseline current, designated as *I*_1_, it is measured as 0.0435 A. During sensor measurement, a voltage of 1 V is applied. The initial resistance *R*_0_, the resistance *R*_1_, and the resistance change ∆*R* are calculated as 31.056, 22.99, and 8.07 Ω, respectively. The measured temperature, denoted as *T*, can be obtained by using the TCR formula, where the TCR is selected as − 0.58%/°C, and the ambient temperature *T*_0_ is 24 °C.

However, the temperature value obtained by the simulation is 68.8 °C, which is far away from the measured temperature (80 °C). In practical applications, temperature measurements are often affected by mechanical stimuli. The temperature response properties of LIG should exclude the influence of pressure variation. Figure S6 shows the relationship between resistance change and temperature for LIG in the pressure range of 0–5 kPa, and the variation of TCR under different pressure loads is shown in Fig. 7c.

To solve the pressure interfaces, a correction factor denoted as α is introduced to revise the resistance changes. When the pressure is below 2 kPa, the correction factor can be calculated by interpolation, as presented in Table 1. For the pressure exceeding 2 kPa, the correction factor remains constantly at 1.31. Considering the correction factor α, the formula for calculating temperature can be modified to

16 $$T = T_{0} + \frac{\alpha \cdot \Delta R}{{R_{0} \cdot {\text{TCR}}}}.$$

**Table 1:** Correction factor for the pressure load

Preload (Pa)	Correction factor *α*
0	1
50	1.10687
200	1.174089
1000	1.300448
≥ 2000	1.31

The modified *T*_*α*_ is calculated as 82.6 °C, which is much closer to the actual cylinder temperature. Thus, the pressure correction factor can be used to reduce the interference of pressure on the temperature measurement.

## Conclusion

A highly sensitive temperature sensing array is prepared by an LDW method, using LIS as electrodes and LIG as temperature sensing layer. Firstly, a FEA photothermal model incorporating phase transition mechanism is developed to investigate the relationships between laser parameters and LIG properties, thereby providing guidance for the laser power (1–5 W) and laser scanning speed (greater than 50 mm/s) during the fabrication process. The electrical properties and temperature responsiveness of LIG are also studied. By changing the laser process parameters, the resistivity of LIG can be regulated within the range of 0.031–-67.2 Ω·m and the average TCR is calculated as − 0.58%/°C. Furthermore, the FEA photothermal model is also used to compare the experiments and simulations data for LIS, and the data exhibit an average difference less than 5%. The LIS sensing samples demonstrate the electrical resistivity of 0.0001–100 Ω·m and show insensitivity to temperature and pressure stimuli. Moreover, as for the LIS-LIG based temperature sensing array, due to disturbance of the LIG temperature sensing by pressure stimuli, by introducing the correction factor, the temperature measurement simulation-experiment difference is decreased from 11.2 to 2.6 °C, indicating good accuracy for temperature measurement.

### Supplementary Information

Below is the link to the electronic supplementary material.

Supplementary file1 (PDF 492 KB)

## Data Availability

The data that support the findings of this study are available from the corresponding author, upon reasonable request.
