# Supplementary material for: All laser direct writing process for temperature sensor based on graphene and silver
Source: Front Optoelectron. 2024 Feb 5;17(1):5. doi: 10.1007/s12200-024-00108-4 (PMC10838876; doi:10.1007/s12200-024-00108-4)
Supplement: Supplementary file 1 — Supplementary file1 (PDF 492 KB) [file 12200_2024_108_MOESM1_ESM.pdf]

Supporting Information

All Laser Direct Writing Process for Laser Induced Graphene and Silver based Temperature Sensor

Qi Li [a], Ruijie Bai [b], Lianbo Guo [c,*], Yang Gao [a,c,*]

[a] Shanghai Key Laboratory of Intelligent Sensing and Detection Technology, School of Mechanical and Power Engineering, East China University of Science and Technology, Shanghai, 200237, China

[b] North Automatic Control Technology Institute, Taiyuan 030006, Shanxi, China

[c] Wuhan National Laboratory for Optoelectronics, Huazhong University of Science & Technology, Wuhan 430074, Hubei, China.

*Corresponding author: Lianbo Guo (lbguo@hust.edu.cn) and Yang Gao (yanggao@ecust.edu.cn)

Keywords: Laser Direct Writing, Temperature Sensor, Finite Element Analysis, Laser induced Graphene, Laser induced Silver

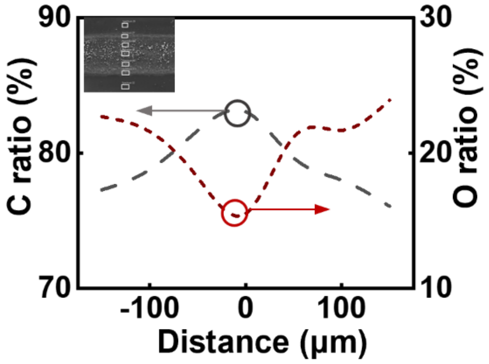

Fig. S1 The C and O element distribution for irradiation area of LIG

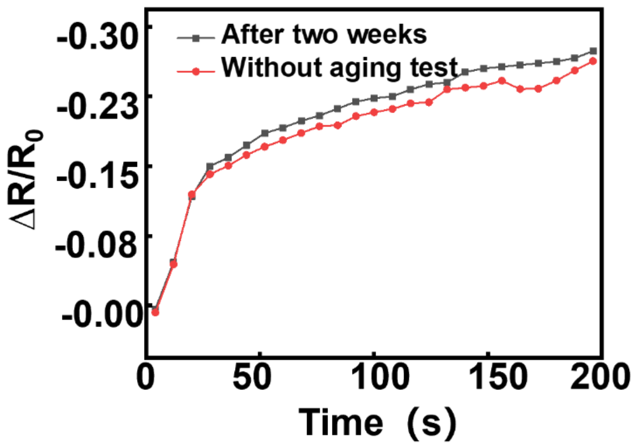

Fig. S2 Temperature response performance of LIG in aging tests

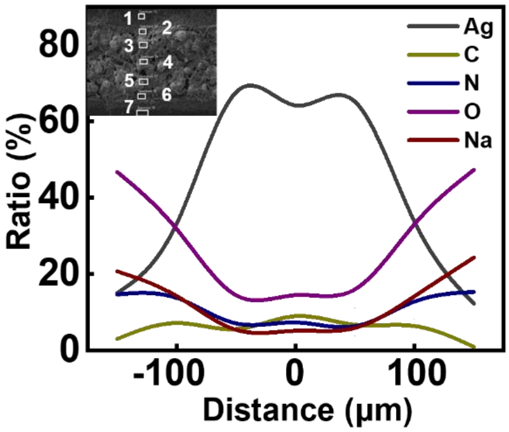

Fig. S3 The EDS spectrum for element distribution of LIS sample

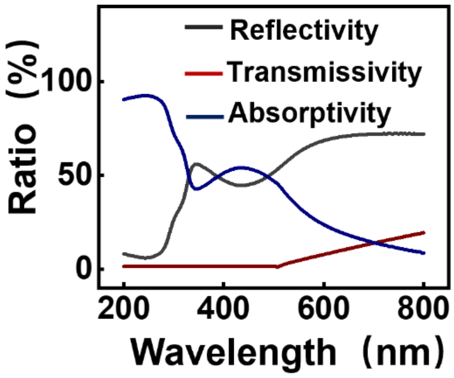

Fig. S4 The optical properties characterization for LIS with different wavelengths, respectively

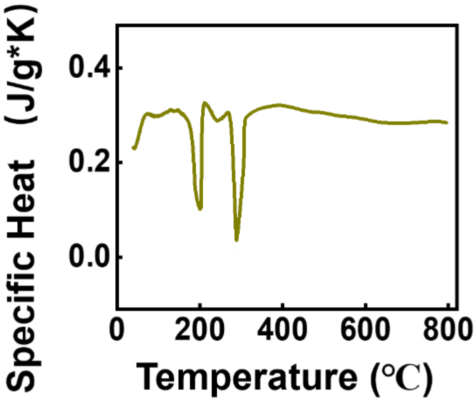

Fig. S5 The specific heat capacity of LIS versus temperature curves

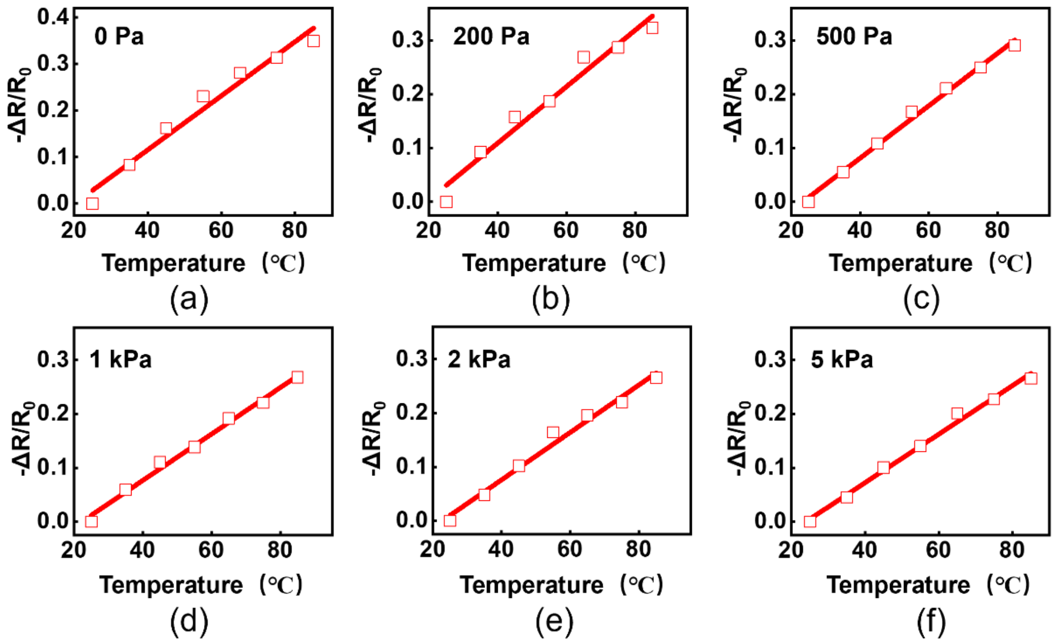

Fig. S6 The relationship between resistance changes and temperature for LIG under the pressure range of 0-5 kPa, respectively

Table. S1 The comparison of temperature sensing performance for LIG with representative examples

Transmission Mechanism	Materials	Sensitivity	Detection range	Figure	Ref.
Resistance	Cardboard	-0.002 (°C^{-1})	18-100 °C	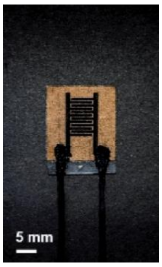	R1
Resistance	PI	~0.035% (K^{-1})	290-350 K	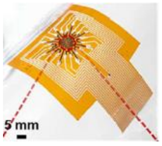	R2
Resistance	PI	−0.04145% (°C^{-1})	30-60 °C	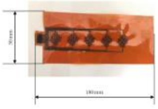	R3
Resistance	PDC (Polymer-derived ceramics)	~−0.55% (K^{-1})	Room Temp to ~450 °C	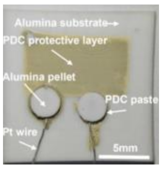	R4
Resistance	Filter paper	-0.28 % (°C^{-1})	10-60 °C	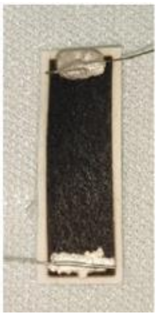	R5
Resistance	Wood cellulose /polyethylene plastic film	(1.25-0.21%) * Temp (°C^{-1})	22-80 °C	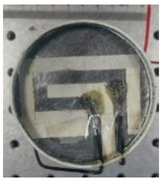	R6
Resistance	CuO NPs	−0.55% (°C^{-1})	20-70 °C	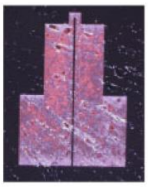	R7
Resistance	GO	−0.512% (°C^{-1})	30-80 °C		R8

Resistance	GO	0.37% (°C^{-1})	30-100 °C	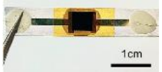	R9
Resistance	F127-resols/V_5S_8	0.0452% (°C^{-1})	10-110 °C	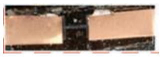	R10
Resistance	PI	0.58% °C^{-1}	25-80 °C		This work

Reference

S1. Ju, K., Gao, Y., Xiao, T., Yu, C., Tan, J. and Xuan, F.: Laser direct writing of carbonaceous sensors on cardboard for human health and indoor environment monitoring. *RSC Adv.*, **10** (32), 18694-18703 (2020)

S2. Xu, K., Li, Q. a., Lu, Y., Luo, H., Jian, Y., Li, D., Kong, D., Wang, R., Tan, J., Cai, Z., Yang, G., Zhu, B., Ye, Q., Yang, H. and Li, T.: Laser Direct Writing of Flexible Thermal Flow Sensors. *Nano Lett.*, **23** (22), 10317-10325 (2023)

S3. Kun, H., Bin, L., Orban, M., Donghai, Q. and Hongbo, Y.: Accurate Flexible Temperature Sensor Based on Laser-Induced Graphene Material. *Shock and Vibration*, **2021**, 9938010 (2021)

S4. Cui, Z., Chen, X., Li, X. and Sui, G.: Thin-film temperature sensor made from polymer-derived ceramics based on laser pyrolysis. *Sens. Actuators, A*, **350**, 114144 (2023)

S5. Kulyk, B., Silva, B. F. R., Carvalho, A. F., Barbosa, P., Girão, A. V., Deuermeier, J., Fernandes, A. J. S., Figueiredo, F. M. L., Fortunato, E. and Costa, F. M.: Laser-Induced Graphene from Paper by Ultraviolet Irradiation: Humidity and Temperature Sensors. *Adv. Mater. Technol.*, **7** (7), 2101311 (2022)

S6. Zhao, Y., Li, C., Qadir, A. and Li, H.: Laser-Patterned Graphite-Based Strain and Temperature Sensor on Disposable Paper Cup. *Adv. Eng. Mater.*, **25** (8), 2201563 (2023)

S7. Mizoshiri, M., Ito, Y., Arakane, S., Sakurai, J. and Hata, S.: Direct fabrication of Cu/Cu2O composite micro-temperature sensor using femtosecond laser reduction patterning. *Jpn. J. Appl. Phys.*, **55** (6S1), 06GP05 (2016)

S8. Silipigni, L., Salvato, G., Fazio, B., Marco, G. D., Proverbio, E., Cutroneo, M., Torrisi, A. and Torrisi, L.: Temperature sensor based on IR-laser reduced Graphene Oxide. *J. Instrum.*, **15** (04), C04006 (2020)

S9. Chen, R., Luo, T., Geng, D., Shen, Z. and Zhou, W.: Facile fabrication of a fast-response flexible temperature sensor via laser reduced graphene oxide for contactless human-machine interface. *Carbon*, **187**, 35-46 (2022)

S10. Yang, L., Yan, J., Meng, C., Dutta, A., Chen, X., Xue, Y., Niu, G., Wang, Y., Du, S., Zhou, P., Zhang, C., Guo, S. and Cheng, H.: Vanadium Oxide-Doped Laser-Induced Graphene Multi-Parameter Sensor to Decouple Soil Nitrogen Loss and Temperature. *Adv. Mater.*, **35** (14), 2210322 (2023)
